# Light-Emitting Channelrhodopsins for Combined Optogenetic and Chemical-Genetic Control of Neurons

**DOI:** 10.1371/journal.pone.0059759

**Published:** 2013-03-27

**Authors:** Ken Berglund, Elisabeth Birkner, George J. Augustine, Ute Hochgeschwender

**Affiliations:** 1 Department of Neurobiology, Duke University, Durham, North Carolina, United States of America; 2 NeuroTransgenic Laboratory, Duke University, Durham, North Carolina, United States of America; 3 Program in Neuroscience and Behavioral Disorders, Duke-NUS Graduate Medical School, Singapore, Singapore; 4 A*STAR/Duke-NUS Neuroscience Research Partnership, Singapore, Singapore; 5 Center for Functional Connectomics, Korea Institute of Science and Technology, Seoul, Republic of Korea; Yale School of Medicine, United States of America

## Abstract

Manipulation of neuronal activity through genetically targeted actuator molecules is a powerful approach for studying information flow in the brain. In these approaches the genetically targeted component, a receptor or a channel, is activated either by a small molecule (chemical genetics) or by light from a physical source (optogenetics). We developed a hybrid technology that allows control of the same neurons by both optogenetic and chemical genetic means. The approach is based on engineered chimeric fusions of a light-generating protein (luciferase) to a light-activated ion channel (channelrhodopsin). Ionic currents then can be activated by bioluminescence upon activation of luciferase by its substrate, coelenterazine (CTZ), as well as by external light. In cell lines, expression of the fusion of Gaussia luciferase to Channelrhodopsin-2 yielded photocurrents in response to CTZ. Larger photocurrents were produced by fusing the luciferase to *Volvox* Channelrhodopsin-1. This version allowed chemical modulation of neuronal activity when expressed in cultured neurons: CTZ treatment shifted neuronal responses to injected currents and sensitized neurons to fire action potentials in response to subthreshold synaptic inputs. These luminescent channelrhodopsins - or luminopsins – preserve the advantages of light-activated ion channels, while extending their capabilities. Our proof-of-principle results suggest that this novel class of tools can be improved and extended in numerous ways.

## Introduction

Control of the activity of defined populations of neurons is a central goal in studies of brain circuitry. Recently various genetic tools have been developed that allow activation or silencing of the activity of targeted neurons in brain tissue, including in live animals (for review see [1]). These approaches can be distinguished according to the effectors/actuators used to control neuronal activity: chemical approaches use small molecules [2], [3], while optical approaches use light [4], [5], [6]. Light-induced (optogenetic) approaches to control of neuronal activity utilize light-activated photosensitive proteins (microbial opsins), such as channelrhodopsins [7], [8], [9], halorhodopsins [10], [11], [12] or archaerhodopsin [13], or operate indirectly via light-activated neurotransmitters or ligands [14], or synthetic small-molecule photoswitches [15], [16]. With the chemical genetic approach, exogenous chemically-activated receptors (such as RASSLs and DREADDs [17], [18], or the Allatostatin receptor [19]) or ligand-gated ion channels (such as Trpv1 and P2X_2_ [14], [20], or LGICs [3]) are expressed in target neurons (for review see [2]). Each of these methods has distinct, complementary advantages. Chemical genetic approaches allow modulation of entire neuronal populations throughout the brain because activation is mediated by a diffusible molecule that can be delivered by systemic application. Unlike optogenetics, chemical genetics is non-invasive because injection of the active molecule does not affect the brain tissue *per se* [20], [21], [22], [23], [24]. However, such approaches typically work over a slow, diffusion-limited time scale. Optogenetic approaches, on the other hand, provide control of neuronal activity with rapid, millisecond time scales [25], [26], [27], [28], [29], [30] and with high spatial precision. However, the need for an external light source limits the number and the location(s) of neurons that can be photostimulated. Because of the small dimensions of optical fibers commonly used for optogenetic photostimulation, as well as attenuation of light in brain tissue due to light scattering, the volume over which neurons can be excited is much smaller than the volume of most major brain structures; this means that only a small fraction of the relevant neurons will be activated. Simultaneous photostimulation of multiple locations can increase this number but requires multiple light sources, which is often impractical. Finally, insertion of light guides or optic fibers to access deep brain structures is technically demanding and causes damage to brain tissue.

Comprehensive interrogation of neuronal circuits requires acute as well as chronic manipulations of spatially defined subpopulations as well as entire populations dispersed over the brain. Thus, there is a need to combine optogenetic and chemical genetic approaches to allow the use of both modes of interrogation in the same brain circuit, and ideally through the same actuator molecule, thereby facilitating the comprehensive study of neuronal systems. For example, such a combination could be achieved by bestowing light-sensitivity on chemical effector-actuator systems. Previous designs towards such combination employed ligand-gated ion channels (Trpv1, P2X_2_), which could be activated either pharmacologically or optically through photolysis of a caged precursor into an active ligand [14]. However, the optical capabilities of this approach are restricted by its use of ultraviolet light (<400 nm), limiting *in vivo* applicability (particularly in the thicker brains of higher organisms), and by the need for the photochemical reaction products to diffuse to the receptors, limiting temporal fidelity, an essential advantage of optogenetics. Alternatively, chemical genetic and optogenetic actuator molecules could be genetically targeted to the same cells, allowing dual mode activation. However, in this case neuronal manipulation is achieved through two different actuators and neurons would be manipulated by two completely different mechanisms.

Ideally, a combined approach that bestows chemical sensitivity on optogenetic probes would be an enormous advantage by permitting acute activation by light, to provide optimal time resolution in defined spaces, and chemical activation, to permit chronic and non-invasive control of entire populations. Here we have designed such a tool for combined chemical genetic and optogenetic manipulation by fusing a luciferase gene [31], [32] to channelrhodopsins. While the channelrhodopsin moiety can be activated directly by light, it can also be activated chemically by the luciferase substrate, coelenterazine. The proof-of-concept studies described here show that light produced by luciferase can activate the linked channelrhodopsin, thereby modulating neuronal activity. The channelrhodopsin moiety also can be activated in a temporally and spatially precise manner by external light. By combining the complementary advantages of both chemical and optical methods, the same genetic construct – termed luminopsin (LMO) - can be used to control neuronal activity via both chemical and optical stimuli. These features make luminopsins useful for the comprehensive interrogation of neuronal circuits.

## Results

Optogenetic control of neuronal activity relies on applying excitation light from an external light source, such as a laser, arc lamp, or light-emitting diode. To extend this approach to include systemic, non-invasive applications, we engineered luminopsins that are channelrhodopsins that produce their own light upon application of a chemical substrate.

### Design of Luminopsins: Luciferase – Channelrhodopsin Fusion Proteins

**Figure 1:**
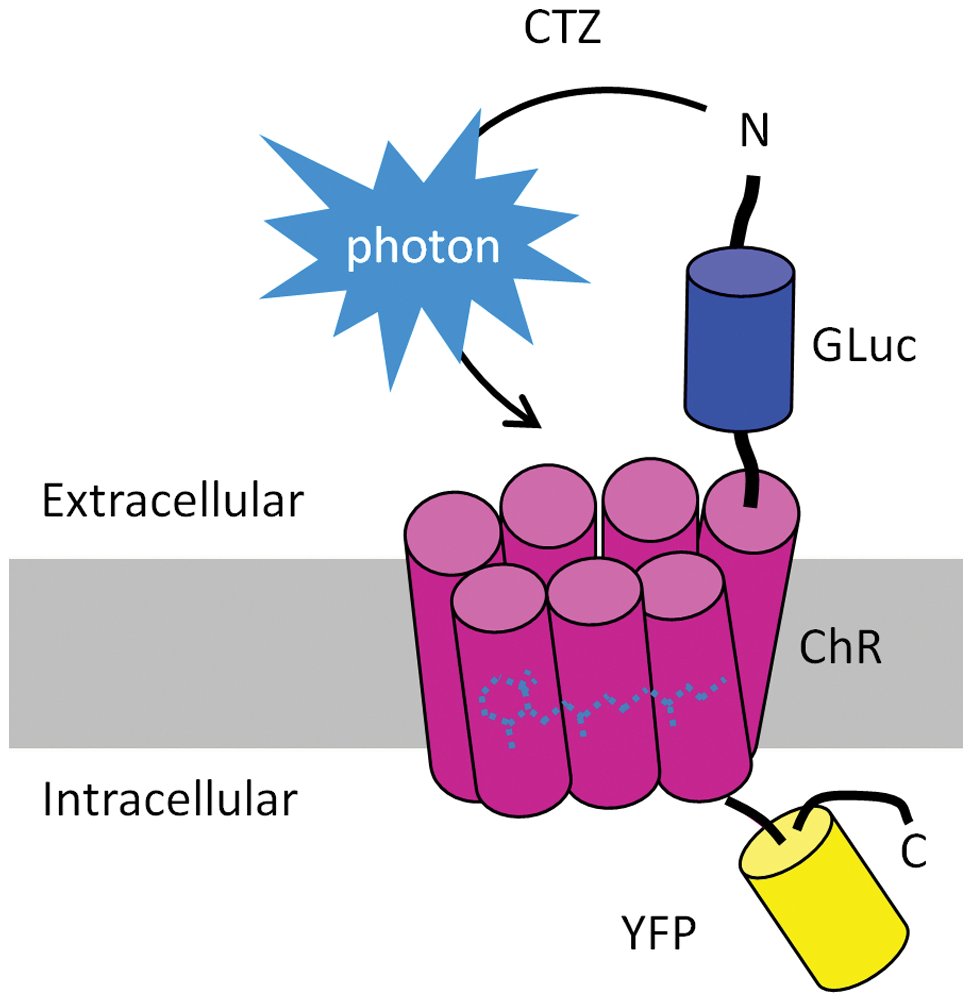
Design of luminopsins, luciferase-channelrhodopsin fusion proteins. Gaussia luciferase (GLuc) is fused to the N-terminus of channelrhodopsin (ChR). Yellow fluorescent protein (YFP) is fused to the C-terminus of ChR. Application of the GLuc substrate coelenterazine (CTZ) leads to an enzymatic reaction resulting in the production of light (photons) and opening of the channel.

Our concept was to create a luminopsin by engineering a chimeric protein that consists of a light-sensitive ion channel, channelrhodopsin, and a light-generating enzyme, luciferase (Fig. 1). We first fused channelrhodopsin-2 from *Chlamydomonas* (ChR2) to a luciferase from the marine copepod *Gaussia princeps* (GLuc). GLuc is the smallest luciferase known (185 amino acids, 19.9 kDa) [31], minimizing possible steric hindrance of the ChR2 component of the luminopsin. The small-molecule substrate, coelenterazine (CTZ), is oxidized by GLuc to generate bioluminescence. Light emission by GLuc is markedly more intense than that produced by other luciferases: the codon-humanized version of GLuc produces 100-fold higher luminescence than firefly luciferase [31], [32]. GLuc emission peaks at 470 nm, which matches the excitation spectrum of ChR2 [7], and its activity is optimal under physiological pH and ionic strength conditions [31]. GLuc is a secreted form of luciferase. It was attached to the N-terminus of ChR2 through a flexible 15 amino-acid linker, so that it would remain outside the cell and available for efficient exposure to CTZ (Fig. 1). The yellow fluorescent protein (YFP) was also added to the C-terminus of ChR2 to provide a fluorescent tag for visualization of the fusion construct [8] (see Materials and Methods). We have named this construct luminopsin-1 (LMO1).

### Functionality of GLuc and ChR2 Moieties within Luminopsin-1

First, we determined whether combining ChR2 and GLuc within LMO1 interferes with the function of either moiety. For this purpose, both PC12 cells and HEK293 cells were transiently transfected with the LMO1 expression construct and then used for experiments after allowing 36 hours for expression of LMO1.

**Figure 2:**
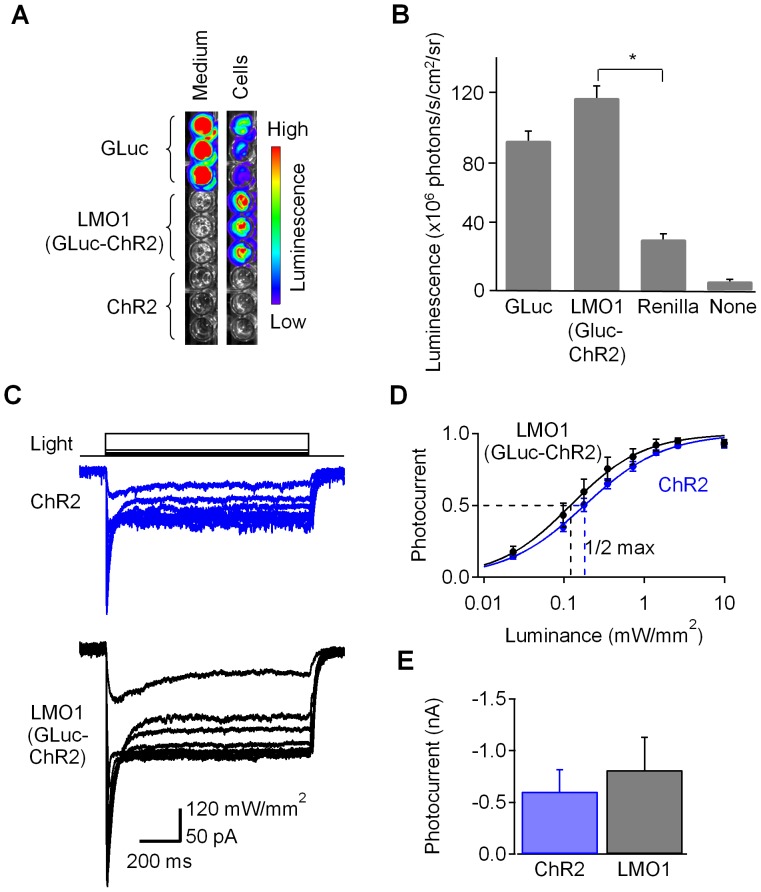
Preserved functionality of individual moieties within luminopsin-1. (A) HEK cells were transfected with native, secreted Gaussia luciferase (GLuc), luminopsin-1 (LMO1; GLuc-ChR2 fusion gene), or with ChR2 alone and bioluminescence was determined by adding CTZ to the medium or to the cells. Only secreted GLuc produced signal in the medium, while bioluminescence generated by LMO1 was found only in cells. (B) Comparison of the bioluminescence signals obtained from cells (10^4^ cells per well, 4 wells per group) transfected with native, secreted GLuc (GLuc), luminopsin-1 (LMO1, GLuc-ChR2 fusion construct), and Renilla luciferase (Renilla) as well as untransfected cells (None). Bioluminescence was comparable between native GLuc and GLuc within LMO1. Luminescence from LMO1 and Renilla was significantly different (*p<0.05; one-way ANOVA followed by Tukey’s test). (C) Comparison of photocurrents induced by physical light in HEK cells transfected with ChR2 (upper panel) and LMO1 (GLuc-ChR2; lower panel). (D) Luminance-photocurrents curves for ChR2 and LMO1 (GLuc-ChR2). When normalized, they showed similar half-maximal luminances (1/2 max). n = 3. (E) No significant differences in maximum photocurrents between ChR2 and LMO1 (GLuc-ChR2) were observed (p>0.05; two-tailed Students’ T-test; n = 3 each). Error bars denote S.E.M in this and subsequent figures.

We quantified the enzymatic activity of GLuc by measuring bioluminescence produced after addition of CTZ [32]. Because native GLuc is a secreted protein, the highest enzymatic activity was found in the culture medium of cells transfected with a GLuc expression construct (Fig. 2A; GLuc – medium). In contrast, in cells transfected with LMO1, luciferase activity was not detected in the culture medium (Fig. 2A; LMO1– medium), confirming membrane-anchoring of the GLuc moiety by ChR2. Cellular luminescence of the LMO1-transfected cells was similar to that of the native GLuc-expressing cells (Figs. 2A and 2B). These experiments confirm that fusion to ChR2 anchors GLuc to the cell membrane and that membrane-bound GLuc retains its enzymatic activity. Thus, anchoring GLuc to the membrane via ChR2 does not interfere with its luciferase activity.

We next assessed whether the presence of GLuc in LMO1 interferes with the light-induced gating of ChR2. To do this, voltage clamp measurements of photocurrents were made in PC12 cells expressing either ChR2 or LMO1. For both constructs, varying the intensity of the excitation light evoked photocurrents of varying magnitude (Fig. 2C). The relationship between light luminance and photocurrent amplitude was determined for each cell and then fit with the Hill equation to quantify the half-maximal luminance (I_1/2_), the Hill coefficient, and the maximum photocurrent (Fig. 2D). None of these parameters was significantly different in comparisons between cells expressing ChR2 and LMO1 (Fig. 2E; for I_1/2_, 186±34 µW/mm^2^ and 141±54 µW/mm^2^, respectively; for the Hill coefficient, 0.89±0.01 and 1.02±0.04, respectively; mean ± S.E.M.; two tailed Students’ T-test; p>0.1; n = 3 each). Thus attaching GLuc does not interfere with the function of ChR2.

### Bioluminescent Activation of Channelrhodopsins

**Figure 3:**
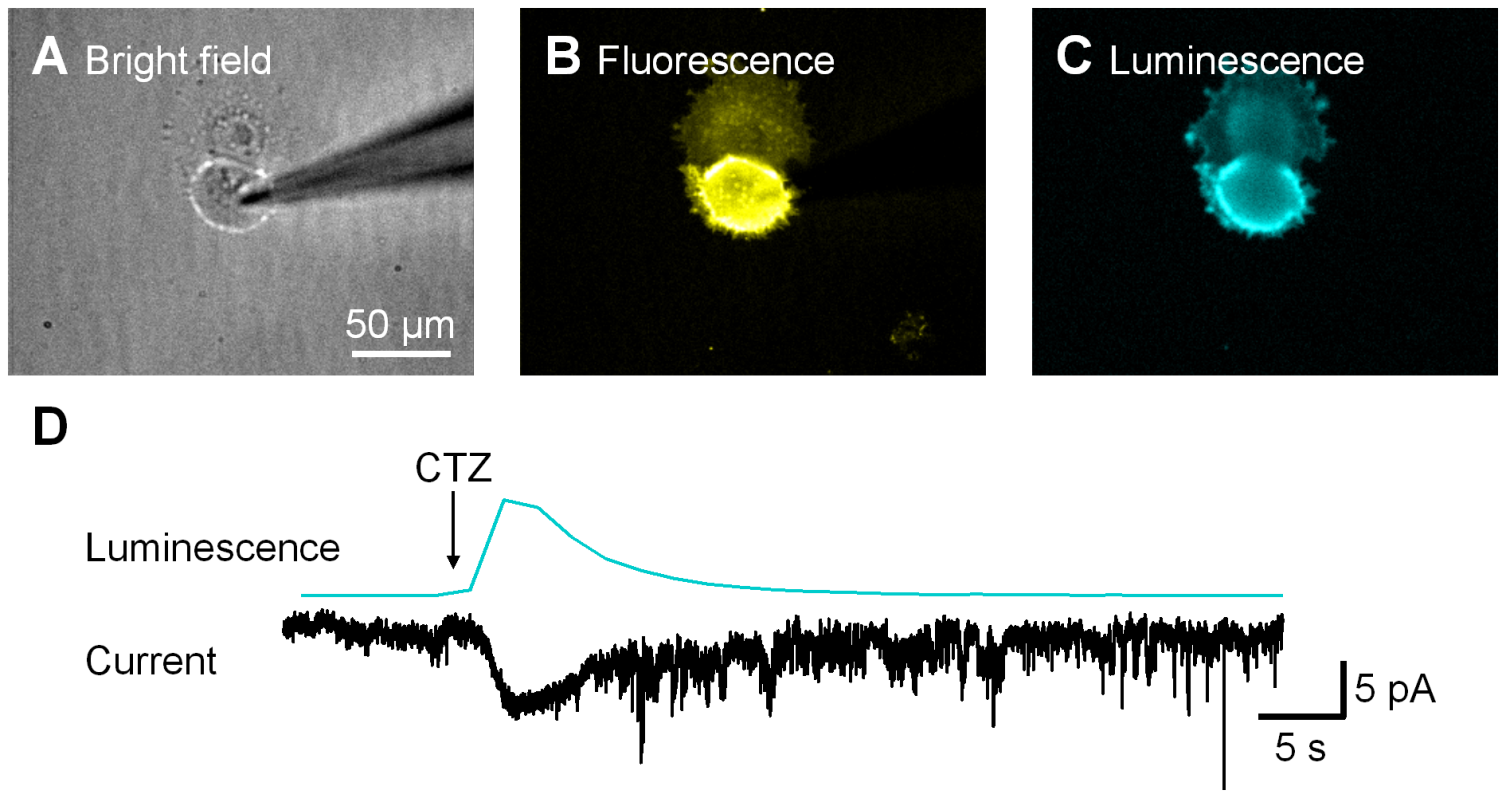
Luminescence activated photocurrent. LMO1 (GLuc-ChR2)-expressing HEK cell (A) was identified by YFP fluorescence (B) and patch-clamped. Coelenterazine (CTZ) application near the cell elicited bioluminescence (C). (D) Luminescence (upper trace) and luminescence-induced photocurrent (lower trace) were recorded simultaneously from the same cell.

**Figure 4:**
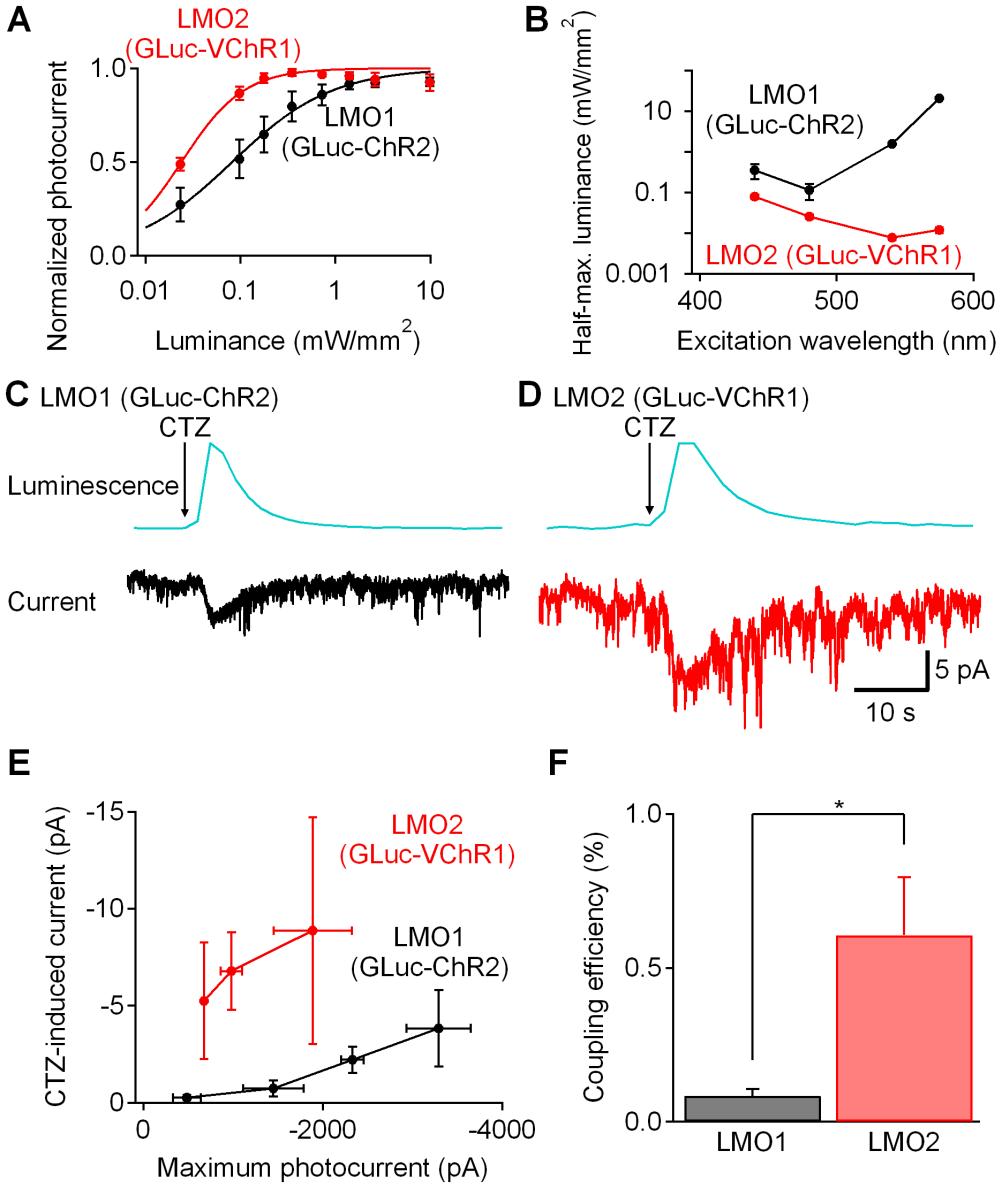
Comparison between luminopsins LMO1 (GLuc-ChR2) and LMO2 (GLuc-VChR1). (A) HEK cells were transfected with LMO1 (GLuc-ChR2) or LMO2 (GLuc-VChR1) (n = 4 and 5, respectively). Photocurrents to various intensities of 470 nm light were recorded and normalized to the maximum to compare light sensitivity between VChR1 and ChR2. The luminance-photocurrent curve of VChR1 is shifted to the left, indicating higher sensitivity of VChR1 to 470 nm light. (B) Half-maximum luminance was lowest at 470 nm for ChR2, and at 540 nm for VChR1, confirming red-shifted excitation spectrum of VChR1. VChR1 showed lower half-maximum luminances at all the wavelengths tested, indicating superior light sensitivity compared to ChR2 (n = 6 for LMO1 and 6 for LMO2). (C and D) CTZ application resulted in photocurrents in cells expressing LMO1 (GLuc-ChR2) (C) and LMO2 (GLuc-VChR1) (D). LMO2 (GLuc-VChR1) showed bigger photocurrent. (E) Correlation between maximum photocurrent induced by an arc lamp and luminescence-induced photocurrent from LMO1 (GLuc-ChR2) and LMO2 (GLuc-VChR1) expressing cells. n = 3 for each point. (F) Coupling efficiency of LMO2 (VChR1-GLuc) was significantly higher than that of LMO1 (ChR2-GLuc) (*p<0.05; two-tailed Students’ T-test; n = 12 and 9, respectively).

Given our findings that the GLuc moiety of LMO1 generates light and that its ChR2 moiety responds to light, we next asked whether ChR2 responds to the light generated by GLuc. We did this by expressing LMO1 in HEK cells (Fig. 3A), with cells expressing LMO1 identified by their YFP fluorescence (Fig. 3B). A brief application of CTZ to these cells, from a nearby pipette, transiently produced bioluminescence emission (Fig. 3C). When CTZ application increased cellular luminescence, a current of similar time course was detected (Fig. 3D). There was a good correlation between the amplitude of this current and the amount of LMO1 expression, as determined by the maximum photocurrent induced by the arc lamp (see Fig. 4F below). As a control, CTZ was also applied to PC12 or HEK cells expressing ChR2 alone. No bioluminescence or inward current was observed in these cells in response to CTZ (Figure S1). These results demonstrate that the bioluminescence generated by GLuc is sufficient to activate nearby ChR2 in LMO1 and establish that this combined optogenetic and chemical genetic approach is feasible.

While the results shown in Figure 3 demonstrate that LMO1 can generate currents, the responses to CTZ generally were small (typically a few pA). To increase the bioluminescence-induced photocurrent, we replaced ChR2 with another channelrhodopsin, *Volvox* Channelrhodopsin 1 (VChR1). VChR1 differs from ChR2 in several aspects: the excitation spectrum of VChR1 is wider and red-shifted, and VChR1-mediated photocurrents have slower kinetics [33], [34]. Despite the red shift, VChR1 is excited strongly by blue light (Supplementary Information in [34]; [35]). To investigate VChR1 as a possible partner for GLuc, we fused GLuc to VChR1 to yield a new probe, termed luminopsin-2 (LMO2). We then compared the performance of LMO2 to that of LMO1 in HEK cells.

YFP fluorescence in LMO2-expressing HEK cells was comparable to that of LMO1-expressing cells, indicating that LMO2 can be over-expressed to a similar level as LMO1. We used photocurrents in response to 470 nm light to compare the light sensitivity of VChR1 and ChR2 in the two versions of luminopsins (Fig. 4A). The relationship between light luminance and photocurrent amplitude was shifted to the left for the case of VChR1, indicating that VChR1 is more sensitive to 470 nm light than is ChR2, even though this wavelength is not at the absorption peak of VChR1. A second comparison of the relative sensitivity of VChR1 and ChR2 to light was obtained by measuring the light luminance required for half-maximal activation of photocurrents (Fig. 4B; Figure S2). For ChR2, this parameter was lowest at 470 nm and increased at wavelengths on either side of the excitation spectrum (Fig. 4B). VChR1 showed its lowest half-maximum luminance at 540 nm (Fig. 4B), confirming its red-shifted excitation spectrum. However, VChR1 showed lower half-maximum values at all the wavelengths tested, indicating the superior light sensitivity of VChR1 in comparison to ChR2. The maximum photocurrents produced by 470 nm illumination, which reflect both expression levels and single channel currents, were comparable between the two (Figure S3). VChR1 has been reported to yield smaller photocurrents compared to ChR2 [35], [36], which is generally attributed to poor membrane expression [37]. Our VChR1 fusion protein contained GLuc, a natively secreted protein, at the N-terminal end, which might be advantageous for membrane trafficking of VChR1. In conclusion, although the red-shifted excitation spectrum of VChR1 does not optimally overlap with the emission spectrum of GLuc, the higher sensitivity of VChR1 to light still makes it a promising candidate as an acceptor of dim light, such as the bioluminescence emission of luminopsins.

To determine whether VChR1 is better activated by GLuc bioluminescence, we applied CTZ to cells expressing LMO2. Overall, CTZ induced larger photocurrents in cells expressing LMO2 (Fig. 4D) than in those expressing LMO1 (Fig. 4C). As judged by fluorescence microscopy, expression levels and targeting to the plasma membrane were comparable for the two LMOs. To account for possible differences in cellular expression level between experiments, we determined the amount of ChR2 or VChR1 expressed by measuring the maximum photocurrent elicited by direct illumination. For both LMO1 and LMO2, the response to CTZ correlated with the maximum photocurrent (Fig. 4E). However, for a given value of maximum photocurrent, responses to CTZ were substantially higher for cells expressing LMO2 than for cells expressing LMO1. This difference was quantified further by measuring the efficiency of coupling between GLuc and the channelrhodopsins. This was calculated by dividing the amplitude of the CTZ-induced current by that of the maximum photocurrent induced by direct illumination of the LMOs. The coupling efficiency of LMO2 was significantly higher than that of LMO1 (Fig. 4F; two-tailed Students’ T-test; p<0.05; n = 12 and 9, respectively). Thus, VChR1 appears to be a better acceptor of bioluminescence from GLuc and this makes LMO2 the better of our two luminopsins.

### Luminopsin-2 Modulates Neuronal Electrical Signaling

**Figure 5:**
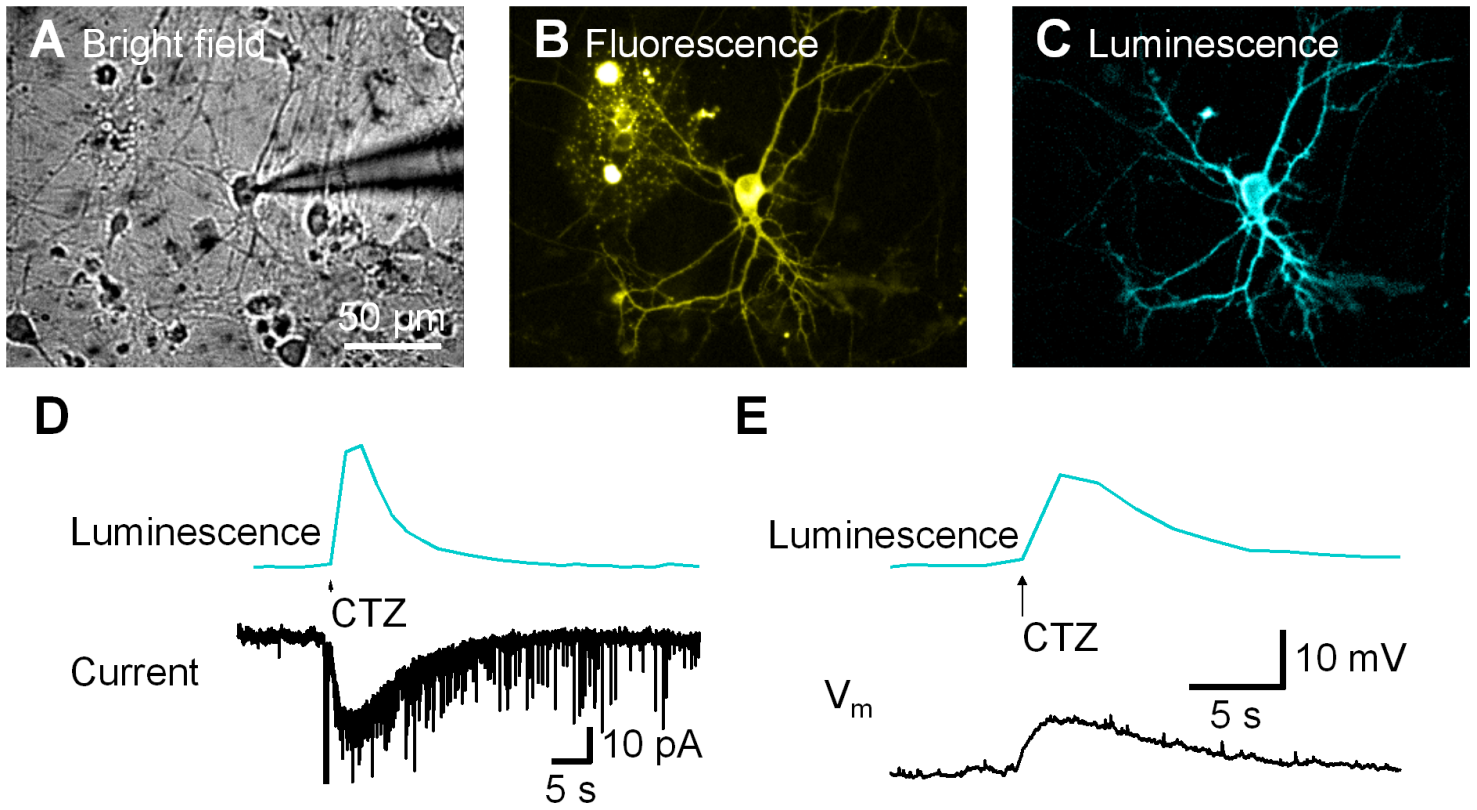
Luminescence-evoked responses in neurons. Hippocampal neurons were transfected with LMO2 (GLuc-VChR1) and patch-clamped (A). Expression of the fusion protein was visualized by YFP fluorescence (B). Upon CTZ application, GLuc generated bioluminescence throughout the processes (C). This bioluminescence evoked both inward current under voltage-clamp (D) and depolarization under current-clamp (E).

To define the ability of LMO2 to alter neuronal activity, this construct was transfected into cultured hippocampal neurons and the electrical and optical responses of the neurons to CTZ application were measured (Figs. 5A and 5B). Upon CTZ application, GLuc generated bioluminescence throughout the processes, indicating relatively homogeneous and high expression of LMO2 on the neuronal membrane (Fig. 5C). This bioluminescence evoked an inward current (Fig. 5D); on average, this current was 10.7±3.4 pA (mean ± S.E.M; n = 8). Under current-clamp recording conditions (Fig. 5E), CTZ depolarized the membrane potential by 3.4±0.9 mV (mean ± S.E.M; n = 8). The coupling efficiency, measured as in Fig. 4F, was 1.7±0.6% (mean ± S.E.M; n = 8). This value is higher than the coupling coefficients measured in HEK cells or PC12 cells, perhaps due to a larger surface area being exposed to CTZ in neurons. Although direct illumination of LMO2 could elicit action potential firing (Figure S4), the depolarization induced by CTZ application was generally small and subthreshold. Thus LMO2 may be more suitable for modulation of neuronal activity, than for direct excitation of neurons.

**Figure 6:**
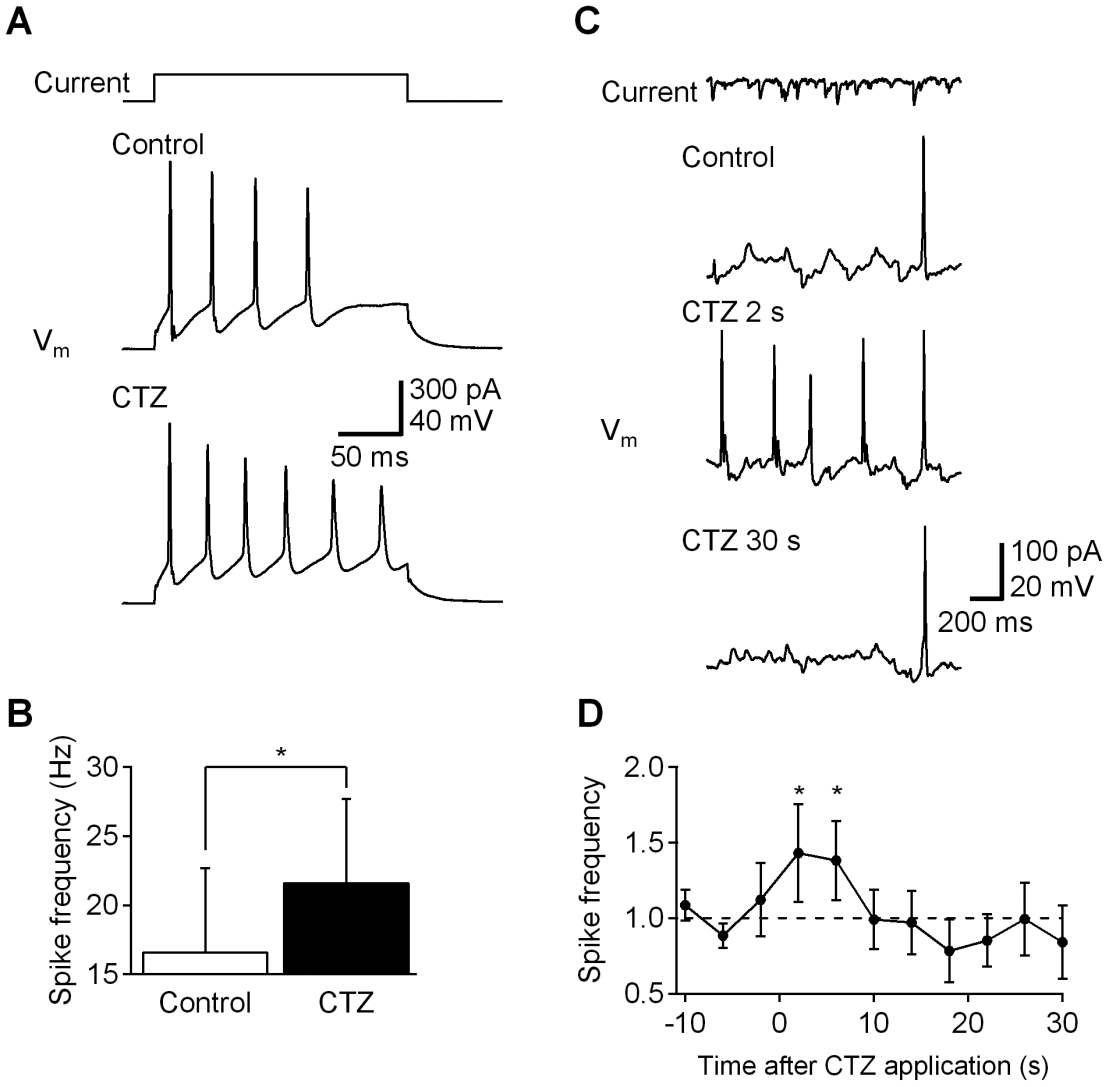
Modulation of neuronal activity by luminescence. Hippocampal neurons were transfected with LMO2 (GLuc-VChR1) and their intrinsic excitability was examined before and after CTZ application. (A) A square pulse (150 pA; top) was injected to a hippocampal neuron under current clamp, eliciting action potential firing (middle). After CTZ application, the neuron fired more action potentials (bottom). (B) Spike frequency with 150-pA current injection was significantly increased by CTZ (*p<0.05; two-tailed paired Students’ T-test; n = 3). (C) Prerecorded subthreshold spontaneous excitatory postsynaptic currents (sEPSCs; top) were injected into a neuron under current clamp, inducing single action potential (control; second from top). After CTZ application action potential firing was increased (CTZ 2 s; third from top) and returned to the baseline (CTZ 30 s; bottom). (D) On average, CTZ application transiently enhanced action potential firing up to 50%; n = 7.

We next tested whether the bioluminescence-evoked depolarization generated by LMO2 could modulate the intrinsic excitability of the cultured hippocampal neurons. First, we asked whether CTZ application altered the ability of neurons to fire action potentials in response to injected current. The neurons fired more action potentials in response to increasing amounts of injected current, with the relationship between injected current and action potential firing saturating at high current intensities. CTZ application caused neurons to fire more action potentials in response to 150 pA of current (Figs. 6A and 6B). Thus, the subthreshold depolarization caused by CTZ activation of LMO2 enhanced neuronal firing and increased the excitability of hippocampal neurons.

To examine the ability of LMO2 to modulate responses to synaptic input, we injected pre-recorded excitatory postsynaptic currents (EPSCs). The mean amplitudes of these currents were adjusted so that they were largely subthreshold, producing approximately one action potential during a 2 s stimulation period in control conditions (Fig. 6C, control). A brief application of CTZ caused the same currents to elicit extra action potentials (Fig. 6C, 2 s CTZ) and action potential firing returned to the baseline level over the next 30 s (Fig. 6C, 30 s CTZ). On average, CTZ application transiently enhanced action potential firing up to 50% (Fig. 6D). The time course of this enhancement coincided with that of bioluminescence emission. Thus, bioluminescence-induced depolarization through LMO2 activation can sensitize neurons to fire action potentials in response to synaptic inputs, making this approach suitable for sensitizing neurons to ongoing synaptic activity.

## Discussion

Optogenetics is a technique that allows acute, “on-or-off” interrogation of defined neuronal populations, while chemical genetic approaches are for chronic and non-invasive modulation of neuronal activity underlying behavioral responses *in vivo*. Comprehensive interrogation of neuronal circuits should integrate the complementary advantages of optogenetic and chemical genetic paradigms, ideally in one genetic construct that can be activated both optically and chemically. We present here proof-of-concept studies for such an approach, specifically fusing *Gaussia* luciferase to channelrhodopsins to create luminopsins. We show that activation of the channelrhodopsin moieties in such fusion proteins can be accomplished either by light delivered from an external light source (the standard optogenetic paradigm) or by light produced by application of substrate to the fused luciferase (a chemical genetic approach). The genetic fusion brings the channelrhodopsin and luciferase moieties within close proximity, allowing light produced by the oxidation of CTZ by luciferase to activate channelrhodopsin. In neurons expressing LMO2, CTZ-induced channel opening yielded subthreshold depolarizations. These responses were able to modulate the intrinsic excitability of neurons, specifically by increasing action potential firing upon injection of current pulses or EPSC-like currents. Thus, cells expressing luminopsins can be activated acutely, although invasively, by light or modulated chronically - and non-invasively - by administering the luciferase substrate CTZ.

Moving the luminopsin concept into *in vivo* applications will be the next logical step. Luciferases and their substrates have been widely used for whole-animal imaging studies; they are nontoxic and well tolerated over repeated applications. Specifically, CTZ has been used as substrate for luciferases and photoproteins *in vivo* in flies, fish, and mice with no side effects reported [32], [38], [39], [40]. For example, after incubating transgenic zebrafish expressing the calcium sensing photoprotein aequorin in CTZ-containing medium freely behaving fish have been monitored for neural activity [39]. In mice, GLuc expressed in the brain produces detectable luminescence through the skull upon intravenous application of CTZ ([32], and our own observations). While conceptually the transfer to *in vivo* studies is straightforward, the specifics of dose requirements and temporal kinetics will need to be experimentally determined.

Because luminopsins can be activated both optically by light and chemically by administration of CTZ, animal models expressing the LMOs will allow defined populations of neurons to be interrogated by both methods and the results from both methods of stimulation can be directly compared and integrated. For example, the role of agouti-related peptide (AgRP) neurons in feeding behavior recently has been investigated in two independent studies: one using a chemical genetic approach and the other using an optogenetic approach [23], [30]. While the chemical genetic study allowed investigation of a variety of aspects of feeding behavior [23], the optogenetic study allowed mapping of neuronal stimulation patterns and behavior [30]. Application of LMOs could allow both types of study to be performed with the same engineered mouse model. More generally, the combination of chemical and optical methodology achieved with the luminopsins results in a synergy that provides dual means of controlling neuronal activity and can yield complementary information that more completely informs the study of neuronal activity and circuitry.

A unique advantage of luminopsins over previous optogenetic or chemical genetic methods for controlling neuronal activity is that activation of the opsin can be simultaneously documented as luminescence. Upon CTZ application to activate neurons, we observed bioluminescence that can be localized and quantified by optical imaging, allowing identification of the neurons that are being activated as well as the extent of their activation. As *in vivo* imaging of bioluminescence becomes increasingly powerful [41], [42], [43], [44], [45], it should be possible to correlate the intensity and location of bioluminescence with the modulation of neuronal activity and with behavioral phenotypes.

The current versions of luminopsins do not consistently drive action potentials in neurons upon CTZ application. However, CTZ induces subthreshold depolarization that alters neuronal excitability and modulates information throughput, allowing tests of the causal relationship of neuronal activity to a given phenotype. For many systems-level and cellular-level processes, subthreshold depolarizations convey physiologically significant information. For example, subthreshold depolarizations are potent for activating synapse-to-nucleus signaling [46], and the relative timing of subthreshold and suprathreshold depolarizations can determine the direction of synaptic plasticity [47]. Subthreshold changes in membrane potential also occur in many sensory receptors, such as photoreceptors and mechanoreceptors. Recognizing the importance of subthreshold stimuli, channelrhodopsin molecules have been mutated to allow subthreshold depolarizations by photostimulation (step function opsins, SFOs [48]), with the goal of generating modulated states of altered excitability. Sensitizing genetically targeted neurons to native, endogenous synaptic inputs can also be achieved by stimulating wild-type ChR2 with lower light intensities or by utilizing different expression levels of ChR2. For example, different transgenic mouse lines expressing the same construct (*ChAT-ChR2-EYFP*) at different levels can be useful for potentiation of basal firing (line 5, [49]) or for precisely timed firing (line 6, [49]). The current version of luminopsin, LMO2, allows both modes of stimulation using the same construct in the same animal.

Nevertheless, it would be beneficial to further improve the performance of luminopsins beyond that achieved by luminopsin-2. Combining light-producing and light-sensing molecules is limited by the rate of photons needed to activate the opsin and by the rate of photon production by the luciferase. While the rate of photons absorbed by channelrhodopsin can be calculated [7], [50], [51], [52], neither the quantum yield of photon emission from Gaussia luciferase nor the distance of the luciferase from the chromophore is known. This makes *a priori* calculations for guiding luminopsin design impractical. Our experiments with LMO1 and LMO2 demonstrate that the photons produced by luciferase-catalyzed CTZ oxidation are sufficient to activate channelrhodopsin. Using these designs as baseline, there are several aspects in which the current luminopsins can be improved. The most important is in the coupling between the light-activated channel and the light emitted by luciferase. There are several ways to improve this coupling. One is to optimize the light-sensitive channel. In the current study we compared luminopsins containing two different channelrhodopsins, ChR2 and VChR1. VChR1 outperformed ChR2 due to its superior sensitivity to light. Recently, several ChR2 mutants have been engineered that remain open for longer periods of time after light activation and show increased sensitivity to low light levels while generating large photocurrents [48], [53], [54], [55], [56]. Luminopsins employing these ChR2 mutants will likely increase the coupling efficiency and thus will increase the potency of the LMOs for chemical genetic modulation. A second strategy is to modify the luciferase. While native Gaussia luciferase generates strong bioluminescence [32], recent mutations of Gaussia luciferase have yielded superluminescent variants with bioluminescence that is 10-fold higher than the native form [57], [58]. These could increase the efficiency of coupling between the luciferase and channelrhodopsins. Finally, we have used native CTZ in our studies and it is possible that analogs of this substrate could achieve even higher light emission and/or coupling efficiency [59], [60].

Luminopsins can be modified to extend their functions in various ways. First, it might be beneficial to incorporate other kinetic variants of channelrhodopsins. Stable step function opsins, SSFOs [5], [37], [53], are to “step” neurons to a stable depolarized resting potential, allowing removal of the light source and initiation of behavioral or physiological experimentation in the complete absence of invasive hardware. Luminopsins would allow similar stepping of membrane potential in a non-invasive way. If a red-shifted firefly luciferase could deactivate SFOs or SSFOs, luminopsin activity could be manipulated in a more controlled manner so that activity is initiated by one substrate (CTZ) and halted by another (beetle luciferin).

Second, the principle of luminopsins may be extended to silence neuronal activity. One such extension would be the use of other microbial opsins, such as the blue-light sensing proton pump from the fungus Leptosphaeria maculans (Mac) or archaerhodopsin from Halorubrum strain TP009 (ArchT) [13], [61]. A luminopsin consisting of a luciferase and a proton pump would allow inactivation of genetically defined neuronal populations.

Third, while in our current studies we fused the luciferase and the opsin in a chimeric molecule, the use of stronger luciferase variants and more sensitive opsins might allow direct expression of each component by different promoters. This would permit reconstitution of luminopsins in neuronal subpopulations defined by the combinatorial expression of two genes, thereby increasing the specificity of neuronal interrogation. This might also allow reconstitution of luminopsins across synapses, analogous to GRASP (GFP Reconstitution Across Synaptic Partners; [62], [63], [64]), thereby enabling functional interrogation of information flow at the level of the synapse.

Fourth, it would be beneficial to use bioluminescence to provide feedback control of neuronal activity. Employing opsins in combination with calcium-sensing photoproteins, such as aequorin, as the light-emitting component will activate the opsin in an activity-dependent manner. Aequorin emits blue light in the presence of CTZ and calcium and thus has been used as a calcium indicator [65], [66], including in transgenic mice [40]. Combining aequorin with a light-sensing opsin such as channelrhodopsin (for activation) or a proton pump (for silencing) would open the possibility of a luminopsin that is controlled by neuronal activity. In fact, combining the third and fourth approaches would provide a new means of connecting neurons beyond chemical and electrical synapses: if luminescence from aequorin were strong enough to go across the synaptic cleft, we could create an “optical” synapse where information is transmitted by photons.

In summary, our proof-of-concept demonstration should stimulate and encourage further developments of the luminopsin concept.

## Methods

### Plasmid Constructs

The following plasmids were used to produce the luminopsins (*Gaussia* luciferase/channelrhodopsin fusions): pCMV-Gluc (Nanolight, cat. no. 202) a plasmid carrying a human codon optimized *Gaussia* luciferase; pcDNA3.1/hChR2(H134R)-EYFP, a plasmid carrying a human codon optimized channelrhodopsin-2 (H134R mutation [67]) gene fused to EYFP (kindly provided by Dr. Karl Deisseroth); and pcDNA3.1/VChR1-EYFP, a plasmid carrying a human codon optimized *Volvox* Channelrhodopsin-1 gene fused to EYFP (kindly provided by Dr. Karl Deisseroth). The luminopsin-1 (LMO1, GLuc-ChR2) and lunimopsin-2 (LMO2, GLuc-VChR1) genes were constructed by inserting GLuc in-frame into the ChR2-EYFP and VChR1-EYFP plasmids at their HindIII and BamHI restriction sites 5′ of the respective channelrhodopsin ATG start codon. The GLuc fragment was generated by PCR amplification of GLuc from pCMV-Gluc with forward primer 5′-TTGTCAAAGCTTGCCACCATGGGAGTCAAAGTT and reverse primers 5′-CGAACGGGATCCCCGTCACCACCGGCCCCCTTGAT (for insertion into ChR2-EYFP) and 5′-TGGCGGATCCGGCAATTCCACCACACTGGACTAGTGGGTCGCCGTCACCACCGGCC CCCTTGAT (for insertion into VChR1-EYFP) and ligation via the HindIII and BamHI restriction sites. The plasmids were amplified and then purified using MaxiPrep kits (Qiagen).

### Cell Culture and Transfection

PC12 [68] (ATCC) or 293T human embryonic kidney fibroblasts (HEK cells [69]) were grown in Dulbecco’s modified Eagle’s medium (DMEM) supplemented with 10% fetal bovine serum, 100 U penicillin and 0.1 mg streptomycin per milliliter, at 37°C and 5% CO_2_ in a humidified atmosphere. For electrophysiology experiments, 3–6×10^4^ cells per well were seeded onto poly-D-lysine (Sigma) coated glass coverslips. For luminescence experiments 1×10^4^ cells were seeded in white 96 well plates (BD Falcon). Cells were transfected using Effectene Transfection Reagent (Qiagen) according to the manufacturer’s protocol. Cells were used for luminescence and electrophysiology experiments 36 hours after transfection. For electrophysiology experiments all-*trans* retinal (1 µM final concentration; Sigma) was added to the cultures the day before the experiment.

Primary neurons were collected from newborn (P0) mice bred at the Association for Assessment and Accreditation of Laboratory Animal Care-accredited animal facility at Duke University or from E18 rat embryos carried by pregnant Sprague Dawley females obtained directly from commercial vendors. All procedures were approved by the Institutional Animal Care and Use Committee at Duke University and followed National Institutes of Health guidelines (Protocol Number: A194-10-08). Animals were euthanized by methods consistent with the recommendations of the Panel on Euthanasia of the American Veterinary Medical Association: female dams were euthanized by isoflurane inhalation, and pups were euthanized by decapitation. Hippocampal neuron cultures were grown on 12 mm poly-D-lysine coated coverslips in 4-well or 24-well plates [70]. Cells were plated in culture medium consisting of Neurobasal Medium (Invitrogen) containing B-27 (Invitrogen), 2 mM Glutamax (Invitrogen), and 5% FCS. The medium was replaced with culture medium without serum the next day. Neurons were transfected on DIV (days *in vitro*) 3 or 4 using Lipofectamine 2000 (Invitrogen) following the manufacturer’s protocol, except that one tenth of the recommended amount of Lipofectamine per well was used. All-*trans* retinal was added to the culture medium the day before recordings. Neurons were used for recording on DIV 7 to 14.

### Bioluminescence Imaging of Cells

The *Gaussia* luciferase substrate, coelenterazine (CTZ), was purchased from Nanolight Technology (P.O. Box 2850, Pinetop, AZ 85935). Coelenterazine free base, the natural form of CTZ found in nature (Nanolight, cat. no. 303 NF-VTZ-FB) was reconstituted in acidified ethanol (5 mg/ml) and diluted in DMEM for culture studies. CTZ was added to cells and/or medium immediately before imaging. Luminescence was measured using an IVIS 100 system (Xenogen IVIS 100, Caliper Life Sciences, Living Image 3.0 software). Images were displayed as a pseudo-color photon count image. Regions of interest were defined using an automatic intensity contour procedure to identify bioluminescent signals with intensities significantly greater than background. The sum of the photon counts in these regions was then calculated.

### Electrophysiology and Optical Methods

Cells were examined on an upright epifluorescence microscope (Eclipse E600-FN; Nikon, Melville, NY) equipped with a 40×0.8 NA water immersion objective, a mercury arc lamp, and an electronic shutter (Uniblitz VS25S; Vincent, Rochester, NY). For YFP fluorescence, Nikon FITC filter cube (excitation: 465–495 nm; emission: 515–555 nm; dichoric: 505 nm) was used. The image was aquired with a cooled CCD camera (CoolSNAP-fx; Photometrics, Tucson, AZ) controlled by RatioTool software (ISee Imaging Systems, Raleigh, NC) and a PC.

For bioluminescence, CTZ (100 µM in the extracellular solution; duration: 100 ms –1 s) was delivered to a cell by puffing, using a glass pipette placed near the cell and a picospritzer (General Valve, Cleveland, OH). Light through 460-nm long-pass dichroic mirror in a Cameleons 2 filter cube (71007a; Chroma, Bellows Falls, VT) was collected at 0.5 Hz with 1.9 s exposure time and 4 by 4 binning. A chamber for the coverslip was constantly superfused with the extracellular solution at ∼500 µl/min.

For electrophysiology, conventional whole-cell voltage- or current-clamp recordings were made using a patch clamp amplifier (Axopatch 1D; Axon Instruments, Foster City, CA) and pClamp 6 software (Axon Instruments). Recording pipettes had resistances of 5–7 MΩ when filled with 140 mM K-gluconate, 2 mM MgCl_2_, 0.5 mM CaCl_2_, 10 mM HEPES, 4 mM Na_2_-ATP, 0.4 mM Na_3_-GTP, and 5 mM EGTA (pH 7.1 titrated with KOH). The extracellular solution consisted of 150 mM NaCl, 3 mM KCl, 2 mM CaCl_2_, 2 mM MgCl_2_, 20 mM d-glucose, and 10 mM HEPES, (pH 7.35, titrated with NaOH).

For routine ChR photoactivation, the FITC filter cube was used. In some experiments, a 430–450 nm excitation filter in Cameleons 2 cube, a 528–553 nm excitation filter in Nikon TRITC filter cube (dichroic: 565 nm), or a 550–600 nm excitation filter in HcRed1 filter cube (dichroic: 610 nm; 41043; Chroma) were also used.

For synaptic current injections, spontaneous excitatory postsynaptic currents were pre-recorded from voltage-clamped Purkinje cells in a mouse cerebellar slice, and the same 1.9 s episode was played back repetitively into a dissociated hippocampal neuron in culture under current clamp. Amplitude was adjusted for each cell so that it would fire one or two action potentials during the 1.9 s period in control condition.

All the experiments were performed at room temperature (21–24°C).

### Data Analysis

All the analysis and statistics were done in Igor Pro 6 (WaveMetrics, Lake Oswego, OR), using in-house and NeuroMatic macros.

## Supporting Information

Figure S1 **CTZ did not cause any response when GLuc was not present.** Even though light from a mercury lamp elicited photocurrent in a PC12 cell expressing ChR2 only (left), CTZ application to the same cell did not induce luminescence or inward current (right).
(TIF)

Figure S2 **Arc-lamp-induced photocurrents of LMO1 and LMO2.** HEK cells were transfected with LMO1 (GLuc-ChR2) or LMO2 (GLuc-VChR1). Photocurrents to various intensities of 4 different wavelengths were recorded. These are the raw data for the analysis in Fig. 4B.
(TIF)

Figure S3 **Maximum photocurrents of LMO1 and LMO2 are comparable.** While LMO2 (GLuc-VChR1) showed lower half-maximum values at all the wavelengths tested, indicating the superior light sensitivity of LMO2 in comparison to LMO1 (GLuc-ChR2), the maximum photocurrents were comparable between the two.
(TIF)

Figure S4 **Direct illumination of LMO2 could elicit action potential firing.** A hippocampal neuron was transfected with LMO2 (GLuc-VChR1) and current-clamped. Light of 470 nm from the arc lamp (blue bar) caused suprathreshold depolarization.
(TIF)
