# Supplementary figures and images for: Light-Emitting Channelrhodopsins for Combined Optogenetic and Chemical-Genetic Control of Neurons
Source: PLoS One. 2013 Mar 27;8(3):e59759. doi: 10.1371/journal.pone.0059759 (PMC3609769; doi:10.1371/journal.pone.0059759)

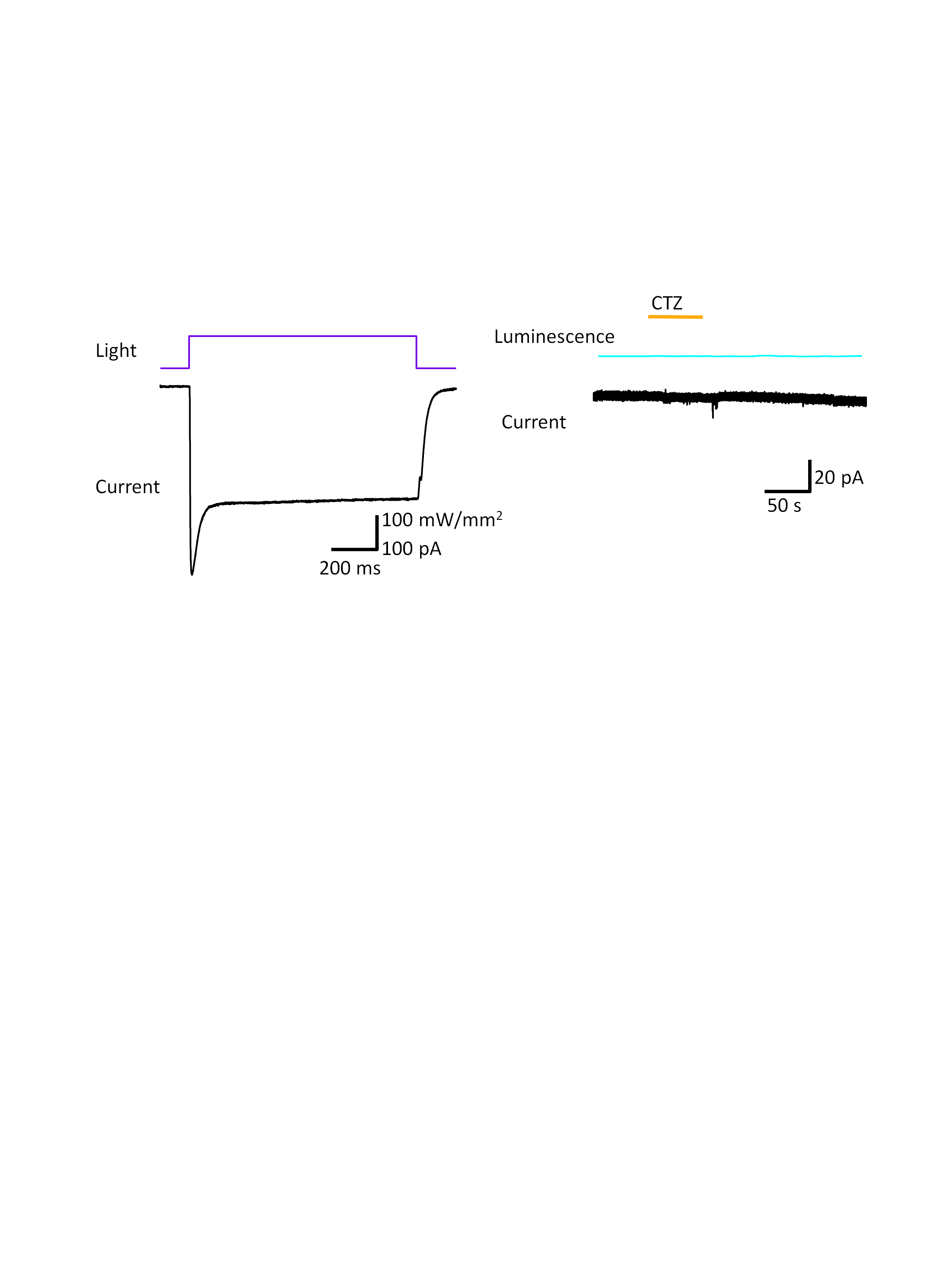

Supplement: Figure S1 — CTZ did not cause any response when GLuc was not present. Even though light from a mercury lamp elicited photocurrent in a PC12 cell expressing ChR2 only (left), CTZ application to the same cell did not induce luminescence or inward current (right). (TIF) [file pone.0059759.s001.tif]

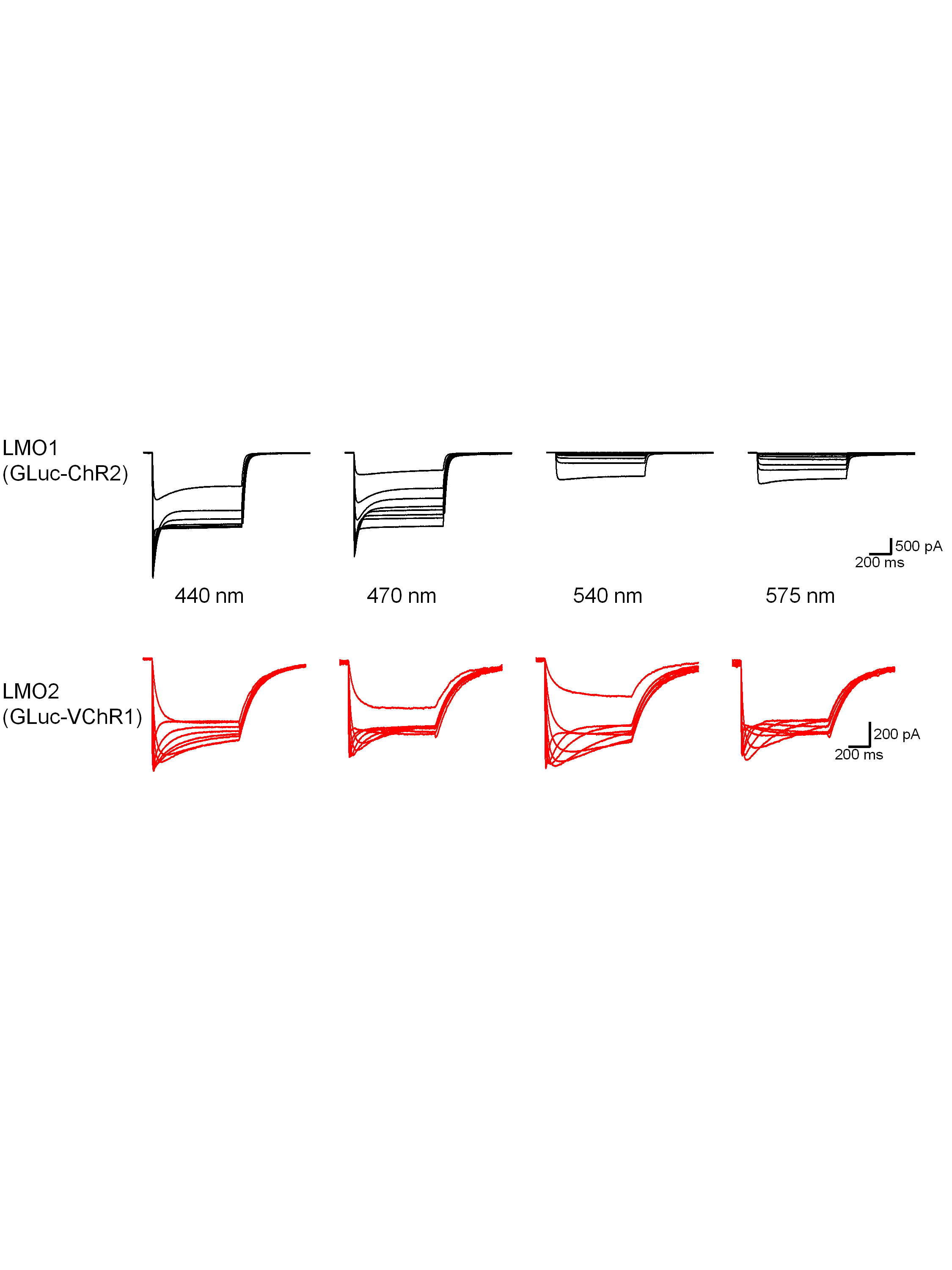

Supplement: Figure S2 — Arc-lamp-induced photocurrents of LMO1 and LMO2. HEK cells were transfected with LMO1 (GLuc-ChR2) or LMO2 (GLuc-VChR1). Photocurrents to various intensities of 4 different wavelengths were recorded. These are the raw data for the analysis in Fig. 4B. (TIF) [file pone.0059759.s002.tif]

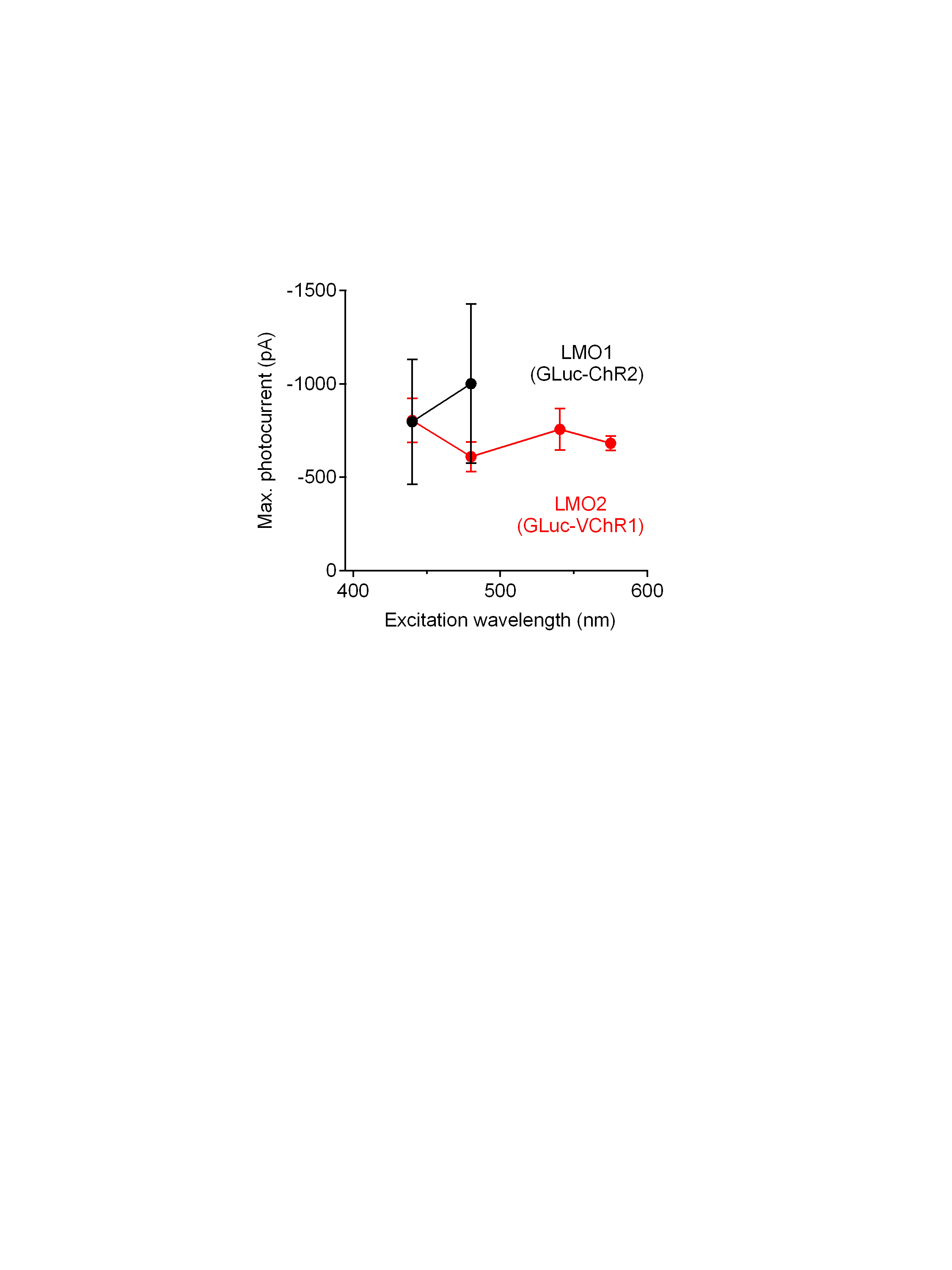

Supplement: Figure S3 — Maximum photocurrents of LMO1 and LMO2 are comparable. While LMO2 (GLuc-VChR1) showed lower half-maximum values at all the wavelengths tested, indicating the superior light sensitivity of LMO2 in comparison to LMO1 (GLuc-ChR2), the maximum photocurrents were comparable between the two. (TIF) [file pone.0059759.s003.tif]

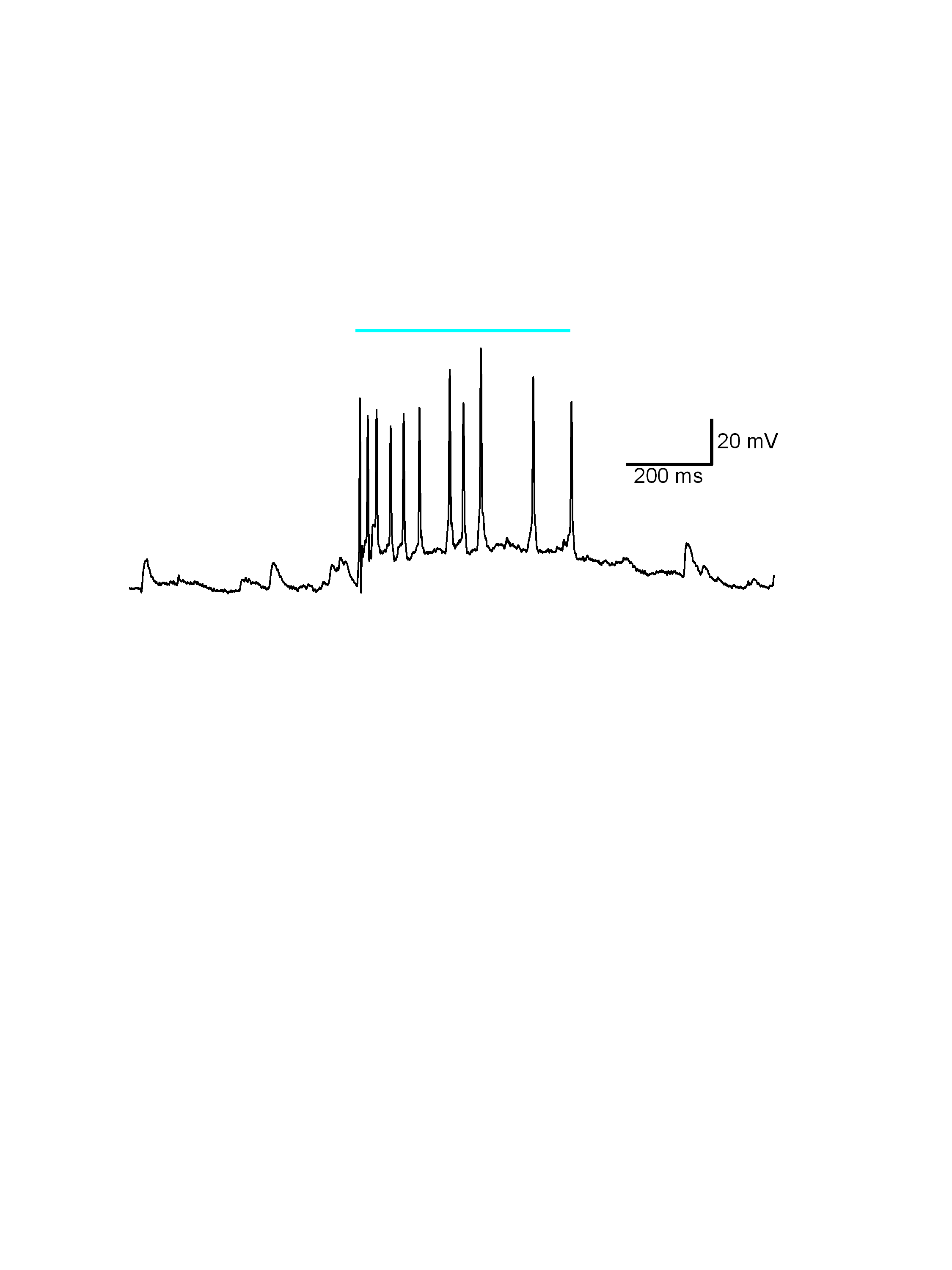

Supplement: Figure S4 — Direct illumination of LMO2 could elicit action potential firing. A hippocampal neuron was transfected with LMO2 (GLuc-VChR1) and current-clamped. Light of 470 nm from the arc lamp (blue bar) caused suprathreshold depolarization. (TIF) [file pone.0059759.s004.tif]
